# Tools for Assessing Neuropathic Pain

**DOI:** 10.1371/journal.pmed.1000045

**Published:** 2009-04-07

**Authors:** Giorgio Cruccu, Andrea Truini

**Affiliations:** 1 Department of Neurological Sciences, La Sapienza University, Rome, Italy; 2 IRCCS San Raffaele Pisana, Rome, Italy

## Abstract

Giorgio Cruccu and Andrea Truini discuss a new pain assessment tool published in *PLoS Medicine* called Standardized Evaluation of Pain and they review other tools to assess neuropathic pain.

According to the latest definition, the term neuropathic pain refers to pain arising as a direct consequence of a lesion or disease affecting the somatosensory system [1].

When physicians and researchers use the term *assessment* of neuropathic pain, they may be referring to two distinct types of assessment: (1) assessing pain intensity and quality and possibly their treatment-induced changes, and (2) diagnosing neuropathic (as opposed to non-neuropathic) pain.

Pain is a complex experience that depends strongly on cognitive, emotional, and educational influences. Hence the pressing need for tools that can measure pain objectively. We distinguish four different levels of “objectivity”: (1) laboratory tests that use quantitative tools and measure an objective response; (2) quantitative sensory testing, a measure that despite using quantitative, graded stimuli inevitably relies on the patient's evaluation; (3) bedside examination, which relies on the physician's experience and the patient's ability and willingness to collaborate; and (4) pain questionnaires, tools that depend entirely on the patient. We review each of these in turn, drawing in part on our previous work in this field [2].

## Laboratory Tests for Diagnosing Neuropathic Pain

Large-size, non-nociceptive afferents (i.e., those that do not carry pain) have a lower electrical threshold than small-size, nociceptive afferents. Unless special techniques are used, i.e., experimental blocks or stimulation of special organs (cornea, tooth pulp, glans), electrical stimuli unavoidably also excite large afferents, thus hindering nociceptive signals. Hence standard neurophysiological responses to electrical stimuli, such as nerve conduction studies (NCS; see Glossary) and somatosensory-evoked potentials (SEPs), can identify, locate, and quantify damage along the peripheral or central sensory pathways, but they do not assess nociceptive pathway function [2], [3].

**Linked Research Article**

This Research in Translation discusses the following new study published in *PLoS Medicine*:

Scholz J, Mannion RJ, Hord DE, Griffin RS, Rawal B, et al. (2009) A novel tool for the assessment of pain: Validation in low back pain. PLoS Med 6(4): e1000047. doi:10.1371/journal.pmed.1000047

Joachim Scholz and colleagues develop and validate an assessment tool that distinguishes between radicular and axial low back pain.

For many years researchers have tried numerous techniques for selectively activating pain afferents. The currently preferred approach uses laser stimulators to deliver radiant-heat pulses that selectively excite the free nerve endings (Aδ and C) in the superficial skin layers. Consensus from over 200 studies now confirms that late laser-evoked potentials (Aδ-LEPs) are nociceptive responses. Late LEPs are the easiest and most reliable neurophysiological tools for assessing nociceptive pathway function and are diagnostically useful in peripheral and central neuropathic pain [4], [5]. In clinical practice, their main limitation is that they are currently available in too few centres [2], [3]. Ultra-late LEPs (related to C-fibre activation) are technically more difficult to record, and few studies have assessed their usefulness in patients with neuropathic pain [6]. Contact heat-evoked potentials are a recent development that still need clinical validation [7].

Painful neuropathies typically and preferentially involve small nerve fibres. Nerve biopsy may be unrewarding in the early detection of small-fibre neuropathy because small-fibre assessment is difficult and requires electron microscopy. Punch skin biopsy can quantify Aδ and C nerve fibres by measuring the density of intra-epidermal nerve fibres (IENF). IENF loss has been shown in various neuropathies characterized by small-fibre axonal loss. Punch skin biopsy is easy to do, minimally invasive, and optimal for follow-up. Despite these advantages, it is useless in central pain and demyelinating neuropathy, and is currently available only in few research centres [8], [9].

## Quantitative Sensory Testing

Quantitative sensory testing (QST) analyses perception in response to external stimuli of controlled intensity (Table 1). Detection and pain thresholds are determined by applying stimuli to the skin in an ascending and descending order of magnitude. Mechanical sensitivity for tactile stimuli is measured with plastic filaments that produce graded pressures, such as the von Frey hairs, pinprick sensation with weighted needles, and vibration sensitivity with an electronic vibrameter. Thermal perception and thermal pain are measured using a thermode, or other device that operates on the thermoelectric effect.

**Table 1:** Summary of choice methods of assessing nerve function per sensation.

Fibres	Sensation	Testing		
		Clinical	QST	Laboratory^c^
Aβ	Touch	Piece of cotton wool	Von Frey filaments	NCS, SEPs
	Vibration	Tuning fork (128 Hz)	Vibrameter^a^	NCS, SEPs
Aδ	Pinprick, sharp pain	Wooden cocktail stick	Weighted needles	LEPs, IENF
	Cold	Thermoroller	Thermode^b^	None
C	Warmth	Thermoroller	Thermode^b^	LEPs, IENF
	Burning	None	Thermode^b^	LEPs, IENF

a Or other device providing graded vibratory stimuli.

b Or other device providing graded thermal stimuli.

c See Glossary.

Modified from [2].

QST has been used for the early diagnosis and follow-up of small-fibre neuropathy that cannot be assessed by standard NCS, and has proved useful in the early diagnosis of diabetic neuropathy. QST is also especially suitable for quantifying mechanical and thermal allodynia and hyperalgesia in painful neuropathic syndromes, and has been used in pharmacological trials to assess treatment efficacy on provoked pains [2].

**Six Key Papers in the Field**

**Treede RD, Jensen TS, Campbell JN, Cruccu G, Dostrovsky JO, et al. (2008) Neuropathic pain: Redefinition and a grading system for clinical and research purposes. Neurology 70: 1630–1635.** [1] The new definition of neuropathic pain and a proposed diagnostic flow-chart that helps to grade neuropathic pain as unlikely, possible, probable, and definite.

**Bennett MI, Attal N, Backonja MM, Baron R, Bouhassira D, et al. (2007) Using screening tools to identify neuropathic pain. Pain 127: 199–203.** [10] A review that brings together the main authors of all the modern screening tools for neuropathic pain and provides a pros-and-cons analysis.

**Chou R, Qaseem A, Snow V, Casey D, Cross JT Jr, et al. (2007) Diagnosis and treatment of low back pain: A joint clinical practice guideline from the American College of Physicians and the American Pain Society. Ann Intern Med 147: 478–491.** These guidelines, specifically devoted to low back pain, face the problem of differentiating nociceptive and neuropathic pain components and recommend adequate methods.

**Rolke R, Baron R, Maier C, Tölle TR, Treede RD, et al. (2006) Quantitative sensory testing in the German Research Network on Neuropathic Pain (DFNS): Standardized protocol and reference values. Pain 123: 231–243.** Although taking 30 min only, this is the most thorough and best validated QST protocol available.

**Dworkin RH, Turk DC, Farrar JT, Haythornthwaite JA, Jensen MP, et al. (2005) Core outcome measures for chronic pain clinical trials: IMMPACT recommendations. Pain 113: 9–19.** A comprehensive review and authoritative consensus that may help the reader to understand the important differences in assessing pain in clinical practice and in pharmacological trials.

**Cruccu G, Anand P, Attal N, Garcia-Larrea L, Haanpää M, et al. (2004) EFNS guidelines on neuropathic pain assessment. Eur J Neurol 11: 153–162.** [2] So far, these are the only existing guidelines for assessing neuropathic pain. They range from bedside examination to the most advanced laboratory tools.

QST abnormalities, however, cannot provide conclusive evidence of neuropathic pain, because QST shows changes also in non-neuropathic pain states, such as rheumatoid arthritis and inflammatory arthromyalgias. QST is time-consuming and thus difficult to use in clinical practice [2].

## Bedside Examination

In patients with neuropathic pain, abnormal sensory findings should be neuroanatomically logical, compatible with a definite lesion site. Location, quality, and intensity of pain should be assessed. Proper assessment requires a clear understanding of the possible types of negative (e.g., sensory loss) and positive (e.g., pain and paresthesias) symptoms and signs. Neuropathic pain can be spontaneous (stimulus-independent or spontaneous pain) or elicited by a stimulus (stimulus-dependent or provoked pain). Spontaneous pain is often described as a constant burning sensation, but may also be intermittent or paroxysmal, and includes dysesthesias and paresthesias. Provoked pains (hyperalgesia and allodynia) are elicited by mechanical, thermal, or chemical stimuli.

Neurological examination in suspected neuropathic pain should include assessment of motor, sensory, and autonomic phenomena in order to identify all signs of neurological dysfunction. Sensory disorders should be recorded in detail, preferably on body sensory maps (neurologists draw the territories where they found a sensory disturbance on schematic charts of the body front and back). Although difficult for the non-specialist and time-consuming for everybody, drawing the sensory abnormality provides valuable information. Tactile sense is best assessed with a piece of cotton wool, pinprick sense with a wooden cocktail stick, thermal sense with warm and cold objects (e.g., metal thermorollers), and vibration sense with a 128-Hz tuning fork (Table 1) [2].

## Questionnaires

Over recent years, several screening tools for distinguishing neuropathic from nociceptive pain have been validated [10]. Some of them, i.e., the Neuropathic Pain Questionnaire (NPQ) [11], ID Pain [12], and PainDETECT [13], rely only on interview questions. PainDETECT was designed to detect neuropathic pain components in patients with low back pain; it has been validated in about 8,000 patients with low back pain, and reaches about 80% sensitivity and specificity [13].

The Leeds Assessment of Neuropathic Symptoms and Signs (LANSS) scale [14] and *Douleur Neuropathique en 4 Questions* (DN4) questionnaire [15] use both interview questions and physical tests (pinprick and tactile hypoesthesia, pain to light touch), and achieve higher sensitivity and specificity than the screening tools that use only interview questions. The higher diagnostic accuracy achieved by the LANSS scale and DN4 questionnaire is hardly surprising given that their scores also reflect physical tests, and emphasizes the importance of clinical examination. Neither LANSS nor DN4 have been specifically evaluated in patients with low back pain, however. Table 2 provides a schematic comparison between all these tools.

**Table 2:** Modern screening tools.

Questionnaires	ID Pain	NPQ	PainDETECT	LANSS	DN4	StEP
**Symptoms reported**						
Ongoing pain						−
Pricking, tingling pins, needles (any dysesthesia)	+	+	+	+	+	+
Electric shocks or shooting	+	+	+	+	+	
Hot or burning	+	+	+	+	+	−
Numbness	+	+	+		+	
Pain evoked by light touching	+	+	+	+		
Painful cold or freezing pain		+			+	−
Pain evoked by mild pressure			+			
Pain evoked by heat or cold			+			
Pain evoked by changes in weather		+				
Pain limited to joints	−					
Itching					+	
Temporal patterns or temporal summation			+			−
Radiation of pain			+			
Autonomic changes	+					
**Physical examination**						
Abnormal response to cold temperature (decrease or allodynia)						+
Hyperalgesia						+
Abnormal response to blunt pressure (decreased or evoked pain)						+
Decreased response to vibration						+
Brush allodynia				+	+	−
Raised soft touch threshold					+	−
Raised pinprick threshold				+	+	+
Straight-leg-raising test						+
Skin changes						−

The minus sign (−) indicates items that reduce the score.

## A New Tool: The Standardized Evaluation of Pain (StEP)

In a new study reported in this issue of *PLoS Medicine*, Joachim Scholz and colleagues present a pain assessment tool called StEP (Standardized Evaluation of Pain) that combines six interview questions and ten physical tests [16]. This novel tool assesses pain-related symptoms and signs and differentiates distinct pain phenotypes reflecting different mechanisms. Besides being diagnostically useful, a standardized approach for differentiating pain phenotypes independently from disease aetiology supports a mechanism-based concept of classifying and treating pain and thus offers an opportunity to improve targeted analgesic treatment [17], [18].

Scholz and colleagues specifically evaluated the diagnostic usefulness of StEP in patients with low back pain, the most frequent (and often challenging) pain condition. Low back pain may comprise both nociceptive axial and neuropathic radicular pain. In these patients, differentiating between nociceptive and neuropathic pain is clinically important because these components require different pain management strategies. The differentiation is also very important for pharmacological trials. By standardizing the assessment of pain-related symptoms and signs, StEP achieves more than 90% sensitivity and specificity in distinguishing neuropathic from nociceptive pain in patients with low back pain (i.e., even better than DN4).

**Glossary**

**Aδ:** Small-myelinated nerve afferents or pathways

**Allodynia:** Pain sensation induced by a stimulus that normally does not provoke pain, and thus implies a change in the quality of a sensation; mechanical allodynia, the most easily tested type, is further classified as dynamic (brush-evoked) or static (pressure-evoked) allodynia

**C:** Unmyelinated nerve afferents or pathways

***Douleur Neuropathique en 4 Questions*** **(DN4):** A tool (“the French”) using seven interview questions and three physical tests

**Dysesthesias:** Spontaneous, non-constant sensations that are clearly unpleasant (e.g., pins and needles)

**Hyperalgesia:** Increased pain response to a stimulus that normally provokes pain (e.g., the pin used in neurological examination)

**ID Pain:** A questionnaire based on six interview questions

**Intra-epidermal nerve fibre (IENF) density:** Measured from punch skin biopsy

**Leeds Assessment of Neuropathic Symptoms and Signs (LANSS):** A tool (“the British”) using five interview questions and two physical tests

**Laser-evoked potentials (LEPs):** Scalp signals evoked by laser stimuli

**Nerve Conduction Study (NCS):** The standard electrodiagnostic tool for assessing peripheral nerve fibre function

**Neuropathic Pain Questionnaire (NPQ):** A tool based on 12 interview questions

**PainDETECT:** A tool (“the German”) based on nine items, including a drawing

**Paresthesia:** Spontaneous, non-constant sensations that are not clearly unpleasant (e.g., tingling)

**Paroxysmal pain:** Sudden, very short-lasting pains (e.g., electric-shock-like sensations)

**Quantitative sensory testing (QST):** A method of assessing sensory function with graded stimuli

**Somatosensory-evoked potentials (SEPs):** Scalp signals evoked by electrical stimuli; the standard electrodiagnostic tool for assessing central somatosensory pathways

**Standardized Evaluation of Pain (StEP):** A new pain assessment tool that uses six interview questions and ten physical tests

The most discriminatory StEP indicators for radicular pain are simple and widely used physical tests such as the straight-leg-raising test (Lasègue's sign), a deficit in cold detection, and a reduced response to pinprick. Although in clinical practice the assessment of sensory abnormalities without graded stimuli increases the variability of outcomes, the standardized application used in StEP improves the diagnostic yield of the straight-leg-raising test and the assessment of sensory deficits, thus suggesting that StEP may be particularly useful in pharmacological trials.

In StEP, unlike the other screening tools and in contrast with commonly held views on neuropathic pain, the descriptors “burning pain” and “brush allodynia” reduce the score for diagnosing neuropathic radicular pain (Table 2). Except for PainDETECT, however, all the other screening tools have been validated in neurological diseases other than spondylotic radiculopathy, a condition that has distinct clinical manifestations. Furthermore, no pathognomonic sensory descriptor exists for neuropathic pain [19]. About 50% of patients with musculoskeletal pain report shooting pain and tingling sensations [20], and 30% of patients with non-neuropathic pain report burning pain [15]. Hence interview questions alone cannot replace clinical examination.

## Conclusion

Despite intensive investigations, the cause of neuropathic pain often remains unknown, and careful assessment is needed before pain can be labelled idiopathic or psychogenic. Pain assessment has advanced enormously over recent years. But whereas the new laboratory tools help in diagnosing neuropathic pain and quantifying damage to the nociceptive pathways, they measure neither pain intensity nor response to treatment. As StEP shows, the most convenient approach is still to combine physical examination and patient's report.

In patients with chronic pain, after years of suffering and frustrated hopes, a psychological component often predominates, making patients non-compliant with new treatments. Ascertaining what is really useful for them becomes at this stage an almost impossible task. Hence we badly need an objective measure of pain intensity and response to treatment. The only method that seems reasonably likely to solve this problem is functional neuroimaging. Yet it will not do so today—only in the future, once it succeeds in providing reliable measures at the individual level and becomes standardized and widely accessible.

## Abbreviations

DN4: *Douleur Neuropathique en 4 Questions*
IENF: intra-epidermal nerve fibre
LANSS: Leeds Assessment of Neuropathic Symptoms and Signs
LEP: laser-evoked potential
NCS: nerve conduction study
NPQ: Neuropathic Pain Questionnaire
QST: quantitative sensory testing: SEP, somatosensory-evoked potential
StEP: Standardized Evaluation of Pain

## References

[1] Treede RD, Jensen TS, Campbell JN, Cruccu G, Dostrovsky JO (2008). Neuropathic pain: Redefinition and a grading system for clinical and research purposes.. Neurology.

[2] Cruccu G, Anand P, Attal N, Garcia-Larrea L, Haanpää M (2004). EFNS guidelines on neuropathic pain assessment.. Eur J Neurol.

[3] Cruccu G, Aminoff MJ, Curio G, Guerit JM, Kakigi R (2008). Recommendations for the clinical use of somatosensory-evoked potentials.. Clin Neurophysiol.

[4] Garcia-Larrea L, Convers P, Magnin M, André-Obadia N, Peyron R (2002). Laser-evoked potential abnormalities in central pain patients: The influence of spontaneous and provoked pain.. Brain.

[5] Treede RD, Lorenz J, Baumgärtner U (2003). Clinical usefulness of laser-evoked potentials.. Neurophysiol Clin.

[6] Truini A, Galeotti F, Haanpaa M, Zucchi R, Albanesi A (2008). Pathophysiology of pain in postherpetic neuralgia: A clinical and neurophysiological study.. Pain.

[7] Granovsky Y, Matre D, Sokolik A, Lorenz J, Casey KL (2005). Thermoreceptive innervation of human glabrous and hairy skin: A contact heat evoked potential analysis.. Pain.

[8] Lauria G, Cornblath DR, Johansson O, McArthur JC, Mellgren SI (2005). EFNS guidelines on the use of skin biopsy in the diagnosis of peripheral neuropathy.. Eur J Neurol.

[9] England JD, Gronseth GS, Franklin G, Carter GT, Kinsella LJ (2009). Evaluation of distal symmetric polyneuropathy: The role of autonomic testing, nerve biopsy, and skin biopsy (an evidence-based review).. Muscle Nerve.

[10] Bennett MI, Attal N, Backonja MM, Baron R, Bouhassira D (2007). Using screening tools to identify neuropathic pain.. Pain.

[11] Krause SJ, Backonja MM (2003). Development of a neuropathic pain questionnaire.. Clin J Pain.

[12] Portenoy R (2006). Development and testing of a neuropathic pain screening questionnaire: ID Pain.. Curr Med Res Opin.

[13] Freynhagen R, Baron R, Gockel U, Tolle T (2006). painDETECT: A new screening questionnaire to detect neuropathic components in patients with back pain.. Curr Med Res Opin.

[14] Bennett MI (2001). The LANSS Pain Scale: The Leeds Assessment of Neuropathic Symptoms and Signs.. Pain.

[15] Bouhassira D, Attal N, Alchaar H, Boureau F, Bruxelle J (2005). Comparison of pain syndromes associated with nervous or somatic lesions and development of a new neuropathic pain diagnostic questionnaire (DN4).. Pain.

[16] Scholz J, Mannion RJ, Hord DE, Griffin RS, Rawal B (2009). A novel tool for the assessment of pain: Validation in low back pain.. PLoS Med.

[17] Attal N, Fermanian C, Fermanian J, Lanteri-Minet M, Alchaar H (2008). Neuropathic pain: Are there distinct subtypes depending on the aetiology or anatomical lesion?. Pain.

[18] Baron R (2008). Mechanisms of postherpetic neuralgia—We are hot on the scent.. Pain.

[19] Hansson P, Haanpää M (2007). Diagnostic work-up of neuropathic pain: Computing, using questionnaires or examining the patient?. Eur J Pain.

[20] Svendsen KB, Jensen TS, Hansen HJ, Bach FW (2005). Sensory function and quality of life in patients with multiple sclerosis and pain.. Pain.

